# Recent Developments in Mechanical Ultraprecision Machining for Nano/Micro Device Manufacturing

**DOI:** 10.3390/mi15081030

**Published:** 2024-08-14

**Authors:** Tirimisiyu Olaniyan, Nadimul Faisal, James Njuguna

**Affiliations:** 1 Advanced Materials Research Group, School of Engineering, Robert Gordon University, Aberdeen AB10 7GJ, UK; t.olaniyan@rgu.ac.uk (T.O.); n.h.faisal@rgu.ac.uk (N.F.); 2 Department of Materials Science and Engineering, Kwara State University, Malete 241104, Nigeria

**Keywords:** ultraprecision machining, sustainable manufacturing, ductile regime machining, minimum quantity lubrication, brittle and hard materials

## Abstract

The production of many components used in MEMS or NEMS devices, especially those with com-plex shapes, requires machining as the best option among manufacturing techniques. Ultraprecision machining is normally employed to achieve the required shapes, dimensional accuracy, or improved surface quality in most of these devices and other areas of application. Compared to conventional machining, ultraprecision machining involves complex phenomenal processes that require extensive investigations for a better understanding of the material removal mechanism. Materials such as semiconductors, composites, steels, ceramics, and polymers are commonly used, particularly in devices designed for harsh environments or applications where alloyed metals may not be suitable. However, unlike alloyed metals, materials like semiconductors (e.g., silicon), ceramics (e.g., silicon carbide), and polymers, which are typically brittle and/or hard, present significant challenges. These challenges include achieving precise surface integrity without post-processing, managing the ductile-brittle transition, and addressing low material removal rates, among others. This review paper examines current research trends in mechanical ultraprecision machining and sustainable ultraprecision machining, along with the adoption of molecular dynamics simulation at the micro and nano scales. The identified challenges are discussed, and potential solutions for addressing these challenges are proposed.

## 1. Introduction

In the manufacturing industry, ultraprecision machining of brittle and/or hard materials has gained popularity for decades. Many of these materials like glasses, plastics, semiconductors, ceramics, and composites have been fabricated through precision/ultraprecision machining processes. Their machining process is referred to as ductile regime machining. Nevertheless, among researchers and machinists, the exact material removal mechanisms underpinning ductile-regime machining of brittle and hard materials (BHMs) remain the subject of debate. This is because ultraprecision machining is complex due to the fact that there is a plethora of interplay of several processes involved. Thus, it is worth having a research tour of this manufacturing process to be able to understand what the trending issues are. Ultraprecision processes are already widely used to improve the performance of automobile and aircraft engines and to produce high-quality surfaces for optical components like lenses and mirrors, thus replacing conventional manufacturing processes [1]. Among these processes are precision/ultraprecision mechanical machining processes. The choice of precision mechanical machining is due to the following: (1) comparative low cost; (2) many complex geometries can be produced; (3) better surface quality and form accuracy.

Materials such as advanced ceramics, composites, semiconductors (silicon), and others henceforth referred to as BHMs have been used in many engineering applications: micro-electro-mechanical systems (MEMS), nano-electro-mechanical systems (NEMS), optoelectronics, optics, electronics, and Information and Communication Technology (ICT). Their unique properties such as wear resistance, high temperature, high thermal strength, and high strength and hardness have made them indispensable as engineering materials [2]. Among the manufacturing techniques through which these materials are incorporated into devices like MEMS and NEMS, especially where complex shapes are involved and better accuracy is required, precision/ultraprecision machining is mostly employed [3,4]. Despite the unique properties of brittle and hard materials (BHMs) compared to alloy metals, machining of these materials poses challenges to the machinists simply because of their high hardness and brittleness coupled with low fracture toughness [5,6]. These challenges include but are not limited to the high cost of manufacturing due to tool wear; poor surface finish; long lead-time; and low material removal rate. Researchers [2,7,8,9,10,11,12] claim that these BHMs can be machined in such a way that their material removal mechanism will be through plastic deformation rather than fracture which results in a crack-free surface—a mechanism referred to as ductile-mode machining. This machining process involves the removal of extremely small amounts of unwanted materials, unlike conventional machining, to achieve an optimal surface roughness of a few nanometres [13,14]. Advances in the precision machining of BHMs have led to the discovery of a “ductile regime” of operation in which the removal of hard-to-machine material is purely plastic. The suppression of a brittle response is desirable in BHMs machining to avoid the generation of deleterious surface flaws [15]. The actual mechanisms underpinning ductile-regime machining of BHMs are not well understood. Thus, although ultraprecision machining is a developed technology, no unarguable consensus has been reached about the exact mechanism of ductile-regime machining of these materials. This is because it involves many phenomenal observations that involve a complex interplay of several processes. Experimentation alone may not be adequate to understand the mechanism behind the machining nature of BHMs due to the intricacy involved when micro-/nanoscale cutting is being carried out. Many of these phenomena have been described and are believed to be observable using molecular dynamic (MD) modelling and simulation. This paper examines the research trends of ultraprecision machining and sustainable ultraprecision machining in addition to the adoption of molecular dynamics simulation. The mechanisms underpinning mechanical ultraprecision machining and identified challenges are systematically analysed, and research gaps and application prospects are summarised.

## 2. Machining Techniques of Micromachining and Nanomachining

**Figure 1:**
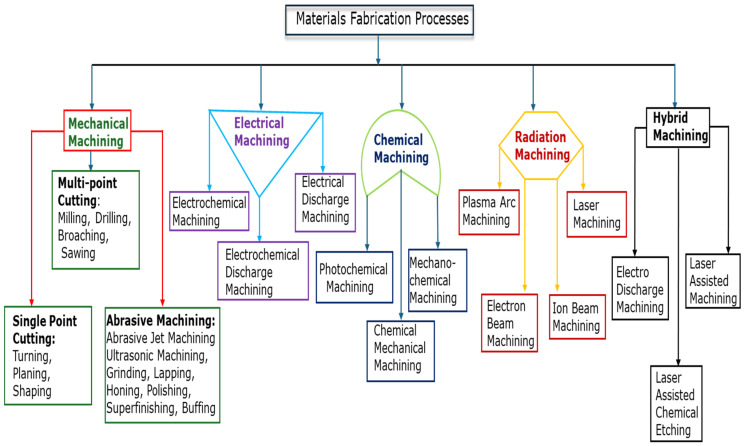
Traditional (conventional) and non-traditional machining techniques.

The various fabrication techniques used in the machining of BHMs and other materials used as components in MEMS, NEMS, and other devices are shown in Figure 1. Hybrid machining is a kind of machining that combines conventional machining with nonconventional machining in which the nonconventional process serves as a source of thermal energy (or as an assistant) to conventional machining like turning, milling, grinding, and so on. The hybrid machining processes listed in Figure 1 are a few of the hybrid machining processes available; others include ultrasonic-assisted machining, plasma-assisted machining, electrochemical grinding (machining), electrochemical discharge machining, and so on [16,17,18,19]. This research work is limited to a brief overview of a few machining processes: single-point diamond turning, precision grinding, precision milling, abrasive waterjet precision machining, laser-assisted machining, and ultrasonic vibration-assisted machining, and they are mostly used in mechanical ultraprecision machining of BHMs. Mechanical ultraprecision machining is universally versatile with a long tradition, since a series of surface structures such as flat, spherical, aspherical, freeform, and others can be generated apart from its usage of processing a large class of engineering materials [20,21].

### 2.1. Ultraprecision Machining

Taniguchi [22] defines ultraprecision machining as machining in which dimensional tolerance is achievable in the order of 0.01 μm and that of surface roughness in the order of 0.001 μm (1 nm). Ultraprecision machining has been described as having originated as diamond machining between the 1950s and 1970s. It was initially designed for metal optics machining at macroscopic dimensions with unachievable tolerances [23]. The precision of tools, machines, and controls in the range of nanometres is remarkably a major prerequisite for ultraprecision machining (UPM). Ultraprecision machining forms part of advanced manufacturing technology in which parts with high accuracy, low subsurface defects, and improved surface quality are generated and meet the requirements of various applications such as optical systems, electronic devices, power devices, and others [8,24,25]. Davies et al. [26] reported by Luo, Goel and Reuben [27] to have quoted a statement in an article in Fortune: “Ultraprecision machining is doing for light what integrated circuits did for electronics”. The invention of integrated circuits (ICs) in 1959 led to the production of more components into which state-of-the-art microchips are fitted according to Moore’s Law, i.e., “The number of components integrated into a semiconductor circuit doubled each year for the first few years of the industry” [28]. However, the doubling occurred nearly every 2 years [29,30]. 

**Figure 2:**
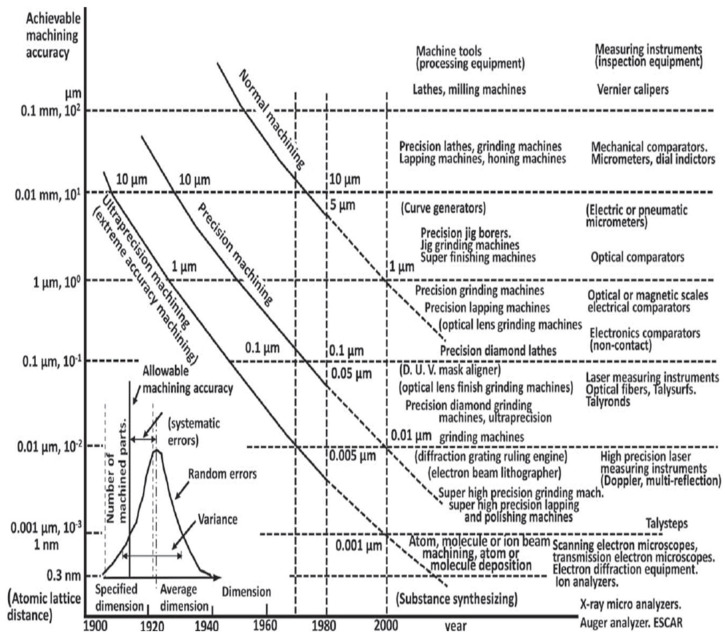
Taniguchi’s chart for the prediction of the development of machining accuracy [22].

From the 1960s–1970s, the technology of ICs was initially and mainly of interest to the military. Nowadays, the technology of ICs is needed in nearly every discipline: telecommunication, medicine, instrumentation and automation, aerospace and aeronautics, computing, modelling and simulation, and so on [29,30]. Likewise, ultraprecision machining was initially designed for metal optics machining with a focus on the energy and defence needs in 1960 [31]. However, the accelerated development of the technology for this machining technique in terms of improved machine design, improved cutting tool design, non-contact drive systems, and computer numeral control (CNC), particularly in the USA, European countries like the United Kingdom and Germany, and Asian countries like Japan and China has led to various applications: X-ray telescope mirrors, infrared reflective optics, annular resonator optics, automotive illumination systems, security and safety, renewable energy generation, and environmental monitoring [24,26,31,32]. With this technology, more components used for MEMS and NEMS devices are developed at a competitive price. It has been claimed that Taniguchi’s chart (Figure 2) is similar to Moore’s law [33] because the main aim of ICs is miniaturisation [24]. How long ultraprecision machining follows suit with Moore’s law is a question that will be left to the researchers in the discipline to answer.

**Figure 3:**
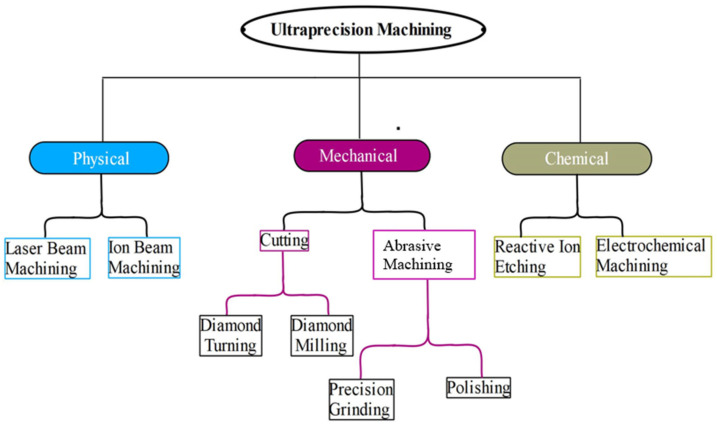
Ultraprecision machining classifications.

Yang et al. [20] classify ultraprecision machining processes based on the material removal process’s physical nature as mechanical, physical, and chemical (Figure 3). Although each classification has its specific area of applications, mechanical ultraprecision machining is universally versatile with a long tradition, since a series of surface structures such as flat, spherical, aspherical, freeform, etc., can be generated in addition to a large class of engineering materials that can be processed [20,21].

### 2.2. Fabrication Techniques in Mechanical Ultraprecision Machining

Traditional (conventional) optical fabrication involves the use of traditional machining processes such as turning, grinding, milling, etc. The final shape and surface of the parts undergo polishing and lapping afterward with an abrasive-loaded lap to achieve the required surface quality. 

Diamond turning is a kind of ultraprecision machining that involves the use of a single-crystal diamond cutting tool in conjunction with CNC precision machines to produce an optical component/surface. It is a fabrication process that is highly technical but deterministic [34]. Unlike the traditional mechanical machining process, polishing and lapping are not needed since the finished surface is very close to the required precision tolerance. The choice of the diamond cutting tool is attributed to the required properties, described as follows: (i) extreme hardness which enables the fabrication of a very sharp cutting edge that results in the production of a fine mirror-like surface at a single pass; (ii) strength and toughness that enable machining of BHMs that are ostensibly believed to be not amenable to machining with conventional tools; and (iii) the precision of the diamond tool coupled with its low wear rate allows surface finishes with tolerance within the precision range to be achieved [35]. Diamond turning process techniques include single-point diamond turning (SPDT), fly cutting technology, slow slide servo technology, and fast tool servo technology, the last three in this list being suitable for complex and aspherical shapes/surfaces. Diamond turning is limited by inherently being able to generate only rotationally symmetrical surfaces [23]. This limitation leads to the other listed fabrication techniques (Figure 1).

## 3. Machining Mechanism of Ostensible Brittle and Hard Materials

Brittle and hard materials such as semiconductors and ceramics are ostensibly believed not amenable to machining because their material removal process to practical forms poses a challenge and is costly. This is a result of their high brittleness and hardness [5]. However, literature has shown that these materials can be machined with low depth of cut, and other parameters are put under control so that a ductile removal mechanism is achieved [24,36]. This is possible because of the development of high-precision machine tools, CNC machines, and control systems [20,21].

### Ductile-Regime Machining (DRM) of Brittle and Hard Materials

The attention of many researchers globally has been drawn to ductile machining of brittle and/or hard materials. This is because they have a series of applications in a variety of fields such as electronics, semiconductors, optoelectronics, information and technology, optical industries, and MEMS and NEMS devices. Suppose certain conditions such as high hydrostatic pressure, depth of cut less than 1 μm, and others are put under control. In that case, there is a possibility that these BHMs can be machined so that their material removal is by plastic flow, leaving a crack-free surface [10,36,37]. The machining process of this kind is called ductile-regime machining.

King and Tabor [38] were the first to observe mechanisms similar to ductile-regime machining of brittle materials when they carried out experimental research on the strength properties and frictional behaviour of rock salt. It is reported that the frictional behaviour of rock salts and other brittle materials is like that of metals where plastic flow is observed instead of brittle fracture. This observation is attributed to the presence of high hydrostatic pressures during sliding [38]. For plastic flow to occur when machining brittle materials at room temperature, high hydrostatic pressure is a prerequisite. This is achieved by using a single-crystal diamond tool having a large rake angle and undeformed chip thickness in the range of ~50 nm [39]. Blake and Scattergood [7] reported that with a large rake angle with a value of −10 to −30 degrees, plastic deformation can be achieved while machining the brittle materials of single-crystal germanium and silicon.

**Figure 4:**
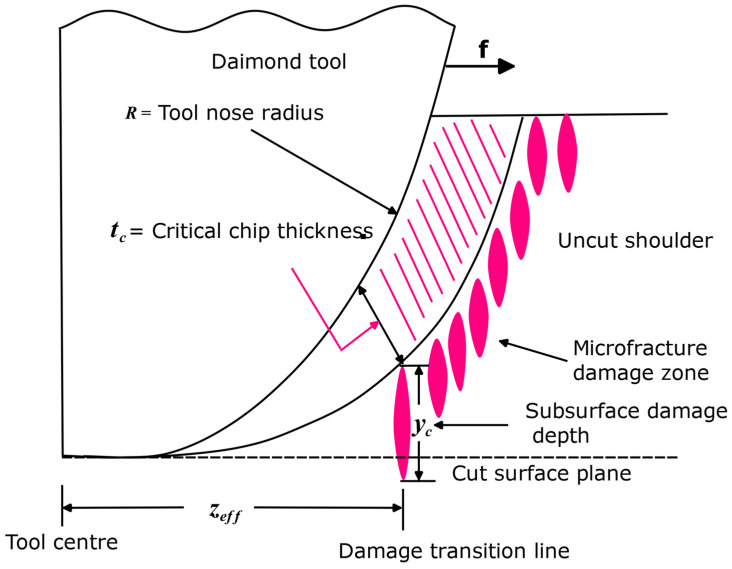
Geometrical machining model: modified from [40].

Equation (1) is a model proposed by Blake and Scattergood [7] as claimed by Blackley and Scattergood [40]. It is made known that it can be used to measure the brittle-ductile transition location while changing tool and machine variables [40]. Regarding the $d_{c}$ value calculated, the model did not explain an apparent change with feed.

(1) $$\;d_{c}=f\left(\frac{Z_{eff}}{R}\right)$$

where $d_{c}$ is critical chip thickness, f is the feed rate, $Z_{eff\;}$ is the distance from the tool centre to the ductile-to-brittle transition line, and R is the nose radius of the tool. The research effort of Blake and Scattergood [7] was complemented by Blackley and Scattergood [40] who put forward a modified model by introducing a new parameter, $y_{c}$, known as subsurface damage depth. This parameter symbolises the depth of the average fracture propagation. This model is represented by Equation (2), and its geometrical machining model is schematically displayed in Figure 4.

(2) $$\frac{Z_{eff}^{2}-f^{2}}{R^{2}}=\frac{d_{c}^{2}}{f^{2}}-2\left(\frac{d_{c}+y_{c}}{R}\right),$$

where R is the tool nose radius, $d_{c}$ is the critical depth of cut, f is the feed rate of the tool, and $Z_{eff}$ is the ductile-to-brittle transition location.

Deterministic processes such as diamond turning and precision grinding are capable of machining brittle materials to produce surface finishes whose characteristics are those attributed to inherently nondeterministic ductile processes like polishing and lapping [2]. It is emphasised that if the depth of cut is small enough, nearly all brittle materials can undergo deformation through plasticity instead of fracture.

When semiconductors are subjected to high pressure under the cutting tool, there is a cause for phase transition from the diamond cubic to the metallic phase (a metastable amorphous phase) while releasing the pressure [15]. This phenomenon in semiconductor research is termed high-pressure metallisation. Plastic deformation experienced during the ductile regime is provided by the metallic phase ductility. Plastic deformation in {111} <110> slip systems during ductile-regime turning of Si is achievable by the phase transformation to an amorphous state [41]. Bifano, Dow, and Scattergood [2] investigated ductile-regime grinding of various varieties of glasses, single crystals, and engineering ceramics (brittle materials). They compared the intrinsic properties of these materials with their grinding ductility and put forward a model to calculate critical cutting depth in Equation (3).

(3) $$d_{c}\;\alpha \;\left(\frac{E}{H}\right){\left(\frac{K_{c}}{H}\right)}^{2}$$

(4) $$d_{c}=A\left(\frac{E}{H}\right){\left(\frac{K_{c}}{H}\right)}^{2},$$

where A the constant of proportionality depending on geometry, machining conditions, and environment, E is elastic modulus, H is hardness, $K_{c}$ is Fracture toughness, and $d_{c}$ is the critical depth of cut, A = 0.15 for some brittle materials such as glasses [2] or A = 6 for metal matrix composite (Al/SiC or Al/Al_2_O_3_) [42].

Recently, the analytical model represented by Equation (4) has been critiqued by Huang et al. [43]. The authors based their argument on the premise that cracks in BHM are atomically sharp rather than having plastic tip zones as used by Bifano et al. [2]. Thus, the familiar form of the relation between fracture energy R and toughness is as follows:

(5) $$R\sim \frac{K_{c}^{2}}{E}$$

With Equation (5), (E/H) will disappear in Equations (3) and (4) because modulus E replaces H. Huang et al. [43] argue that the calibration to arrive at the proportionality constant A is based on a limited set of brittle materials, and (E/H) is limited to a narrow range of 13–17 considering a large number of brittle materials. In addition, the variability of parameters in Equation (4) is of concern because their values depend on the kinds of tests used in their determination, and the sensitivity of these parameters is influenced by factors like composition, microstructure, crystallography, and so on. An analogous equation (or amended model) to Equation (4) is written as follows:

(6) dc=8.7HE12KcH2,

Comparison and predictions of $d_{c}$ are reported using Equation (4) with A = 0.15 and Equation (6) for various BHMs [43]. However, the authors warn that the users of the two analytical models should be extremely careful. This is because, apart from the materials’ properties, other factors such as tool shape and machining conditions are sensitive to the cutting depth. These factors are explicitly excluded in both models.

**Figure 5:**
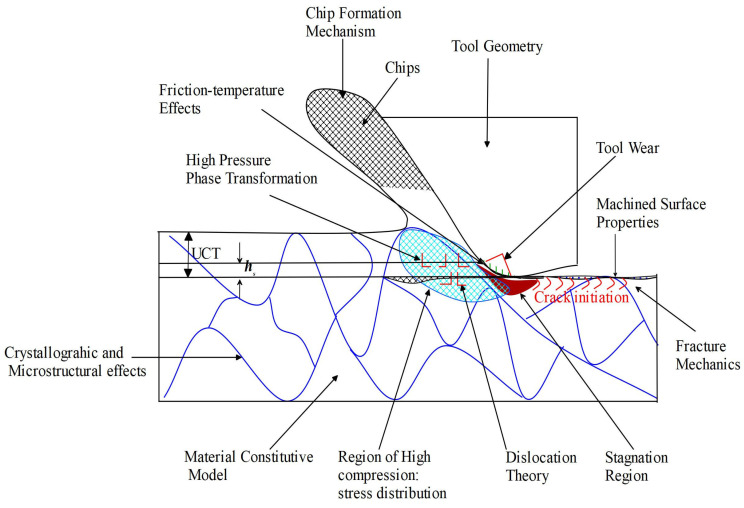
Cutting of brittle and/or hard materials at the nanoscale: complex phenomena involved, redrawn and modified from [58].

Huang et al. [43], Leung et al. [44], and Huang et al. [45] affirm that despite several attempts made by machining researchers and experts to have a better understanding of brittle materials’ ductile behaviour, no unarguable consensus has been reached regarding the basic physical mechanisms behind the ductile-regime machining of these brittle materials. This is corroborated by the fact that micro/nanometric cutting of BHMs displays so many phenomenal observations that involve a complex interplay of several processes (Figure 5). The recent publications indicate that the research is still ongoing in this research area [34,35,36,37,38,39,40,41,46]. Even though much investment and research effort has been made on silicon and germanium being the most focused materials at the beginning of ductile-regime machining invention and research, and micro-components were majorly given attention in the semiconductor and information and communication technology (ICT) sector, issues of tool wear and improvement on their machinability are still ongoing investigations [47,48,49,50]. Applications of ultraprecision/precision machining have now extended beyond the semiconductor and the ICT sectors. Ultraprecision/precision machining is now a manufacturing process for fabricating many components in MEMS/NEMS devices; defence; aerospace and aeronautics; surveillance devices; optoelectronics; imaging/medical diagnosis; and so on [26]. Other materials like ceramics (SiC), organic materials such as polymers, polycarbonate, and polystyrene, and semiconductors (gallium arsenide) have been investigated and research is going on to either determine their machineability or improve their machineability through precision/ultraprecision machining [51,52,53,54,55,56,57]. The overall purpose of this research is to ensure a lower cost of fabrication by trying to surmount one or more challenges associated with mechanical ultraprecision machining as stated herein.

The theory of ductile machining contends that all materials, including brittle materials, will transform from brittle to ductile during machining in an area below a critical cutting depth. It is believed that the energy required for crack propagation exceeds the energy required for plastic deformation below the critical cutting depth. Therefore, the primary method of material removal at this site is plastic deformation [25].

## 4. Recent Developments in Mechanical Ultraprecision Machining of Brittle and/or Hard Materials

There have been various investigations carried out on mechanical precision or ultraprecision machining of brittle and/or hard materials in the last three decades. Issues of tool wear, the surface quality of the machined surface, and the cost-effectiveness of the mechanical precision machining processes are still the challenges that the researchers in this discipline are trying to find a lasting solution to [9,51,54,55,59,60,61,62,63,64,65]. These mechanical precision machining processes can be multi-point cutting, single-point cutting, or abrasive machining. The investigations and research carried out on most of these processes are analysed and reported in the following sections.

### 4.1. Single-Point Diamond Turning of Brittle and/or Hard Materials

Single-Point Diamond Turning (SPDT) has its origin in the USA in the late 1950s with the primary aim of satisfying the demands in aerospace fields and national defence. Originally, SPDT was used to machine metals such as copper and aluminium, but with tool geometry redesign, improved precision machines design, and the micro-dimensional size of diamond cutting tools, SPDT is now being used to machine other materials such as plastic materials (PMMA) for lenses, ceramics (like silicon carbide, gallium arsenide), semiconductors (like silicon, germanium), composite materials, and ferrous metals like steel [21,66,67]. Twentieth-century precision engineering requires fast production as well as better surface quality for optical devices. This demand is met by SPDT as one of the manufacturing techniques [64]. It is affirmed that SPDT technology enables the study of machining at cutting depth values down to the atomic-scale range [59].

Ravindra and Patten [64] investigated the SPDT of quartz to assess its machineability via ductile-regime machining. In the research, the surface quality of quartz for mirror and window applications as well as tool wear was investigated. It was possible to produce surface roughness (Ra) values of less than 45 nm without subsurface damage. It should be noted that this was achieved with a single-diamond tool with a high negative rake angle of 45°. It has been reported that the negative rake angle of the tool is necessary because it provides the needed hydrostatic pressures that enable plastic deformation of the workpiece material beneath the tool edge radius [25]. SPDT successfully resulted in an improved surface finish of quartz without causing any visible surface or subsurface damage. However, the cutting tool was not free from being worn. It was concluded that SPDT ductile mode machining is feasible for quartz material.

Chen et al. [55] investigated the mechanical properties of GaAs through indentation tests with attention to anisotropic machinability. This technique is similar to SPDT through an assumption that the diagonals of the indenter acted in a similar way to the cutting edge of a diamond tool with a negative rake angle. It was reported that ductile machining of GaAs with better cutting performance was achieved with a diamond tool with a large negative rake angle (−40°), and a critical depth of cut 26.57 nm was achieved in the hardest cutting direction. In the ductile-regime diamond turning of GaAs, the material removal mechanism is thought to be plastic deformation driven by high-density dislocations, and a smooth surface at the nano scale was successfully formed along all the orientations on the (001)-oriented GaAs [55].

**Figure 6:**
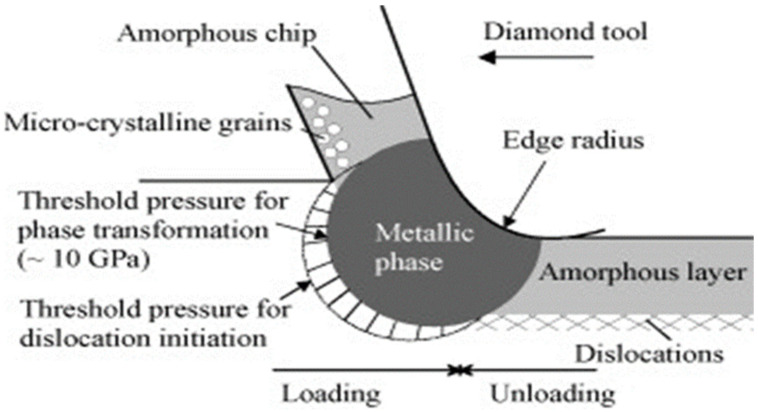
Schematic model for subsurface damage mechanism in silicon during ductile machining [73].

Heidari et al. [68] carried out experiments and finite-element simulation to investigate the effects of tool rake angle and nose radius on the surface quality of ultraprecision diamond-turned porous silicon. Their investigation involves the use of a diamond tool with different negative rake angles. It is reported that if the nose radius of the tool is bigger, the brittle fractures are suppressed around the pore edge, and an improved surface quality can be achieved. This is premised on the choice of optimal tool geometry. The examination of the ductile-machined surfaces using Raman spectroscopy as reported by Heidari et al. [68] reveals that the amorphisation of the surface layer became more important when either the tool rake angle decreases or the tool nose radius increases. As opposing what is observable with non-porous silicon cutting, the cutting pressure of porous silicon is reduced as the rake angle decreases: there is an occurrence of more brittle fractures around the pores that release pressure. Similarly, Jasinevicius et al. [69] also reported the amorphisation of silicon when it was diamond-turned and examined through a transmission electron microscope (TEM). Ductile-regime machining of silicon was obtained before the fracture onset. This ductile regime was attributed to a phase transformation. The phase transformation is reported to have been evidenced indirectly by the detection of the amorphous phase in the machined surface. Jasinevicius et al. [69] concluded that the mechanisms of chip formation are plastic deformation and transformation shearing and this was a result of an intermediate pressure/stress metallic phase transformation in the material. It was reported that TEM analysis of ductile chips revealed that no sign of dislocation activity or crystalline phase was observed. An investigation by Fang et al. [70] affirms this claim by reporting that silicon chips have an amorphous structure when cutting is in ductile mode but have a polycrystalline structure when cutting is in brittle mode. Contrary to this observation are the reports by Goel et al. [71] and Wang et al. [72] that reveal that ductile chips contain a polycrystalline structure. Contrary observations by these authors were affirmed by the investigation carried out by Yan et al. [73] who proposed a subsurface damage model (Figure 6) under high-pressure conditions by considering silicon’s phase transformation and dislocation behaviour. They concluded that the cutting chips are a combination of an amorphous phase and a polycrystalline phase. Nevertheless, it should be noted that this observation is for the chips. For the machined surface, for instance, with silicon and silicon carbide, the deformation of these materials and other BHMs has been attributed to (1) phase transformation as a result of the presence of high hydrostatic pressure and (2) mobility of dislocation [70,72,73,74,75]. The deformation through phase transformation is the most widely reported mechanism by much of the literature [64,70,72,74,76]. Some investigations by some researchers reveal that, in addition to phase transformation, dislocation mobility contributes to the material removal mechanism of BHMs [72,73,77,78]. The majority of researchers attribute the material removal mechanism of BHMs at the nanoscale to the extrusion process. However, Wang et al. [72] numerically associate the material removal mechanism of silicon with extrusion and shear. It is claimed that the chip structure observed by the silicon is because of the cooperation of extrusion with shearing that led to final material removal. The author emphasized that the shear process is depressed when the ratio of the tool edge radius to the undeformed chip thickness is high.

### 4.2. Ultraprecision Grinding of Brittle and/or Hard Materials

**Figure 7:**
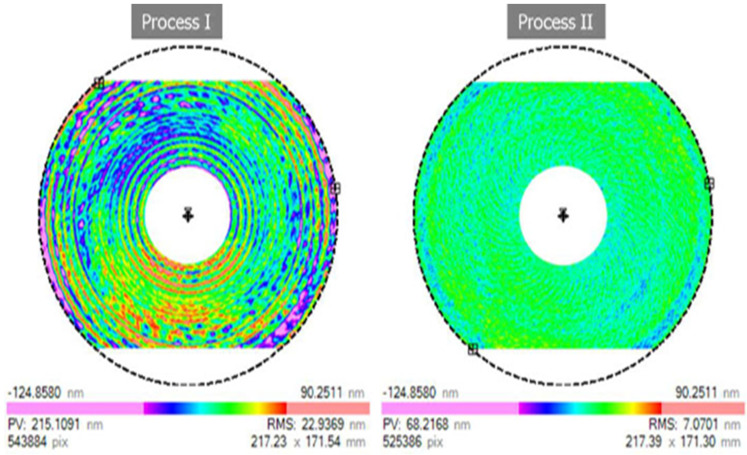
Interferometer measurements of surface form error after fine correction. Process I: grinding, polishing and smoothing, and fine correction; process II: grinding, ultraprecision grinding (UPG), polishing, and fine correction [79].

**Figure 8:**
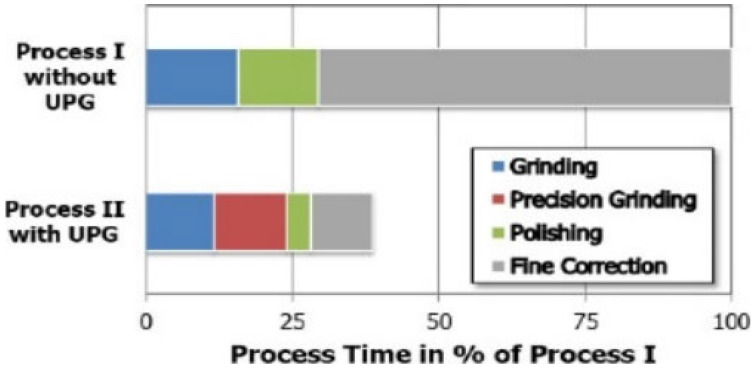
Comparison of relative process times of process chains I and II split into the respective process steps [79].

Investigation by King and Tabor [38] reveals that it is possible for a brittle material (rock salt) to undergo grinding in a ductile manner. Rumpel and Enderli [79] investigated and compared asphere surface productions of Zerodur^®^ asphere in terms of process time and surface figure errors via (I) the industry-standard process chain, which involves grinding, polishing and smoothing, and fine correction; (II) the proposed production chain, which involves grinding, ultraprecision grinding (UPG), polishing, and fine correction. The authors claim that the second route of production that includes UPG before polishing can serve as a possible alternative to diamond turning where it is not a suitable machining technique such as in optical glasses. The surface form errors in terms of surface roughness of the two production routes after the final fine correction processes are shown in Figure 7. It was concluded that employing UPG achieved aspheres of superior quality with an irregularity of 68 nm and an RSM of 7 nm (Figure 7). Likewise, unlike production route (I), process times for polishing and fine correction are reasonably lower which leads to less overall production time (Figure 8). Although process II is being used in the authors’ company for volume production of lightweight aspheres, they assume lesser use of process II by many companies due to the highly demanding process control in UPG.

The investigation by Tao et al. [80] indicates the non-availability of estimation methods and prediction models for the surface topography of ground silicon wafers considering the material removal characteristics between the grains and silicon. A novel three-dimensional topography modelling framework from a micro-scale perspective is proposed. The results from the simulation and experiment reveal that the grinding parameters and the distance from the wafer centre have the most effects on assessment indices: groove angle (*β*) and three-dimensional surface roughness (*Sa*). With feed rate having no significant influence on (*β*) and (*Sa*) in the ductile regime, it is reported that when wheel speeds increased, (*β*) and (*Sa*) declined but increased with higher wafer speed. However, at a higher feed rate in a brittle mode, *Sa* increases. It is concluded that to have a reduced roughness or a smooth wafer surface and improved grinding accuracy, the selection of small grain size grinding wheels, increasing wheel speed, and reducing the wafer speed are essential.

Ultraprecision grinding experiments were carried out by Feng et al. [81] to investigate the material removal characteristics of reaction-bonded silicon carbide (RB-SiC) and pressureless sintered silicon carbide (S-SiC). Cup wheels of different grain size were employed ranging between #120 and #12,000, and grinding grooves as well as both surface topographies and surface morphologies were measured to reveal the material removal mechanism. The two materials display distinct material removal characteristics, and for most high-performance SiC ceramic products, #2000 diamond wheel is recommended. With a #2000 diamond wheel, a surface roughness of about 3 nm and a groove depth of less than 8 nm are obtainable for both RB-SiC and S-SiC ceramics. These values are reported to fulfil the requirements of most high-performance applications of SiC such as aircraft engine components, extrusion dies, brake disks in the automobile industry, propulsion units, and so on [81].

**Figure 9:**
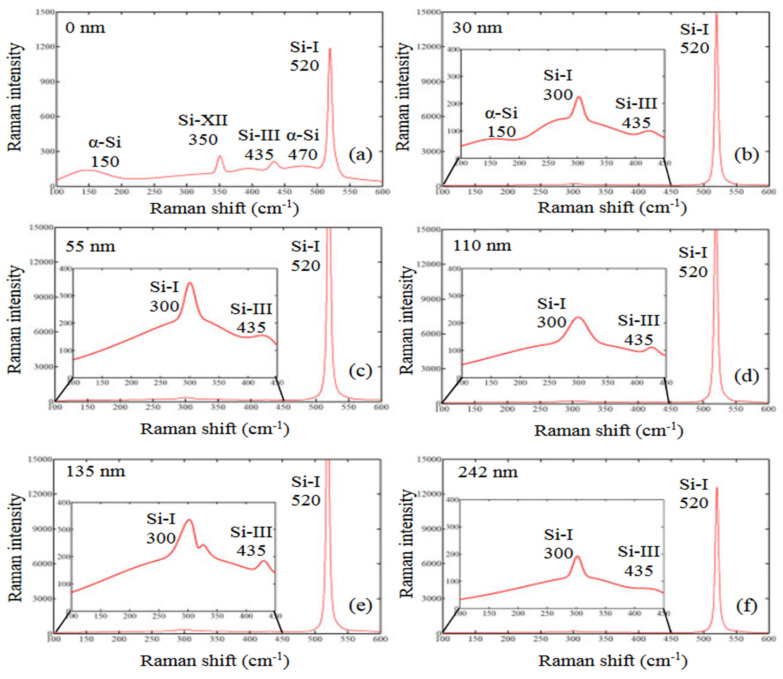
Raman spectroscopy examination of the finely ground silicon wafer at etching depths of (**a**) 0 nm, (**b**) 30 nm, (**c**) 55 nm, (**d**) 110 nm, (**e**) 135 nm, and (**f**) 242 nm [82].

An experimental and simulation investigation was carried out by Chen et al. [82] on the generation and distribution of residual stress during nano-grinding of monocrystalline silicon. Their study reveals that the silicon wafer surface material undergoes phase transformations with an observation of amorphous silicon (a-Si), Si-I, Si-III, and Si-XII at an etching depth of 0 nm–30 nm based on the Raman spectroscopy examination (employing a stepwise wet etching method). However, beyond this range of values, no a-Si was observed (Figure 9). It is stated that phase transformation could lead to a change in material volume and residual stress can be induced by an inhomogeneous change in volume. At a greater etching depth, the stress transitions from compressive to tensile. The key mechanism for residual stress generation during the nano-grinding of silicon according to [82] is the phase transformation and its accompanying volume changes.

Various research has been carried out on the precision grinding of brittle/hard materials and its mechanisms. A comprehensive review of precision grinding and its mechanism for brittle or hard materials can be found in [45,83,84].

### 4.3. Ultra-Precision Milling of Brittle and Hard Materials

Research on ultraprecision milling of brittle and hard materials is sparse in the literature. Cheng et al. [85] carried out an investigation to analyse nano-surface generation in ultra-precision raster milling (UPRM) theoretically and experimentally. With UPRM, it is possible to fabricate non-rotational optical surfaces with form accuracy at a sub-micrometre level and surface finish at the nanometric range without demanding further post-polishing [85]. An investigation by Sun et al. [86] indicated the novelty of ultra-precision side milling (UPSM) by pointing out challenges associated with mechanical and non-mechanical fabrication/generation of hybrid micro-optics from infrared (IR) materials such as silicon (Table 1).

Considering the high form accuracy in addition to low processing time, it has been argued that it is challenging to achieve the one-step generation of hybrid IR freeform micro-optics that display a complex freeform primary surface with the imposition of high-frequency diffractive secondary micro/nanostructures via the major known non-mechanical and mechanical machining methods [86]. It is claimed and affirmed experimentally that this is possible using UPSM in ductile mode [86].

### 4.4. Abrasive Waterjet Precision Machining

Unlike the mechanical ultraprecision machining techniques of hard and brittle materials discussed previously, abrasive waterjet precision machining (AWPM) has the following advantages: (1) materials independence; (2) structural and chemical integrity of the parent material is maintained; (3) no generation of heat-affected zones or induction of any heat damage to the parts; (4) with a single tool, multiple machining modes; cutting, milling, bevelling, and drilling among others can be carried out; (5) faster cutting; and (6) difficult and delicate materials like hardened steel, composites, alloys, laminates, and so on can be machined [87]. Liu [87] compared abrasive waterjet (AWJ) machining with other precision machining methods considering various factors such as tool performance comparison, particle size and thickness, and types of materials among others to list these advantages of AWJ. Despite these advantages, AWJ has its limitations when machining composites; the two partner materials may be weakened by abrasive garnet or bonding between the fibres and matrix may be affected. This could lead to a reduction in the mechanical properties or a reworking time increase [88].

**Figure 10:**
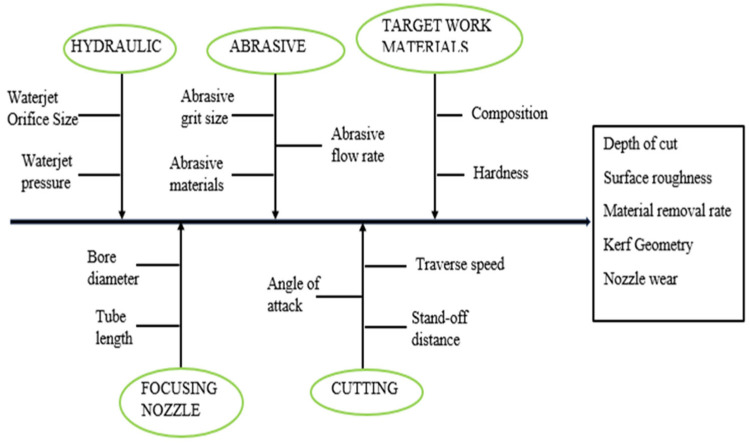
Process parameters and output parameters of AWJ [90].

Zhao and Guo [89] investigated the surface topography and microstructure of the cutting surfaces machined by AWJ. It is reported that materials machined with AWJ displayed no heat-affected zone on the machined surface unlike the surface obtained by wire electro-discharge machining. Based on the various materials used, it is concluded that with hard materials a smooth machined surface is obtained with ease, while serious erosions are observed on soft material surfaces. Some processing parameters need to be put into consideration when AWJ is being carried out (Figure 10).

How these processing parameters affect the targeted output parameters can be obtained from a review by Korat and Acharya [90]. The survey of AWJ shows that it is a major machining technique for hard materials such as composites, steel, and most ceramics. For an in-depth understanding of the mechanisms of AWJ and various types of AWJ, interested readers are referred to excellent reviews in the literature [87,90,91].

### 4.5. Heat-Assisted Machining of Brittle and Hard Materials

There are hybrid machining techniques that are employed using conventional machining processes such as turning, milling, grinding, and drilling and various sources of heat to preheat BHMs or during machining of BHMs at the macro-/microscale level. Such processes include plasma-assisted machining; laser-assisted machining; induction-assisted machining; flame-assisted machining; and others. Any heat source is expected to meet requirements such as high heat energy density to ensure that the material is rapidly preheated; easy control of the heated area’s size and location; easy and safe integration to the convectional machines; and the cost should be reasonable enough to implement [92]. Based on these requirements, laser source has been recommended as a machining method capable of producing high-quality machined parts with cost-effectiveness [92,93]. Although the investment cost of laser may be high, its many applications or benefits override the cost. Among these benefits are very high power intensity, easy integration into conventional machines, the laser beam can be focused to ensure that heat concentration is high, coherence, and so on [94] cited [95].

Various scientific investigations have been carried out to rise to the challenges stated earlier [96,97,98,99,100,101]. They claim that heat-assisted machining provides a better surface finish, reduction in thrust force, better MRR, tool life longevity, and increased cutting depth. Heating locally a brittle material with an intense source of heat, such as laser or plasma, there is a reduction in the material’s yield strength and its value is below its fracture strength. Thus, ductile material removal by a cutting tool prevails instead of brittle fracture [102].

#### 4.5.1. Laser-Assisted Machining (LAM) of Brittle and Hard Materials

**Figure 11:**
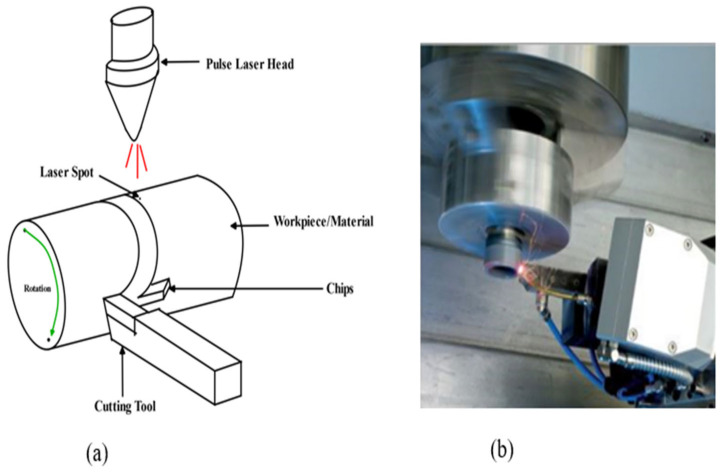
Schematic LAT (**a**) and experimental set-up of LAT (**b**).

**Figure 12:**
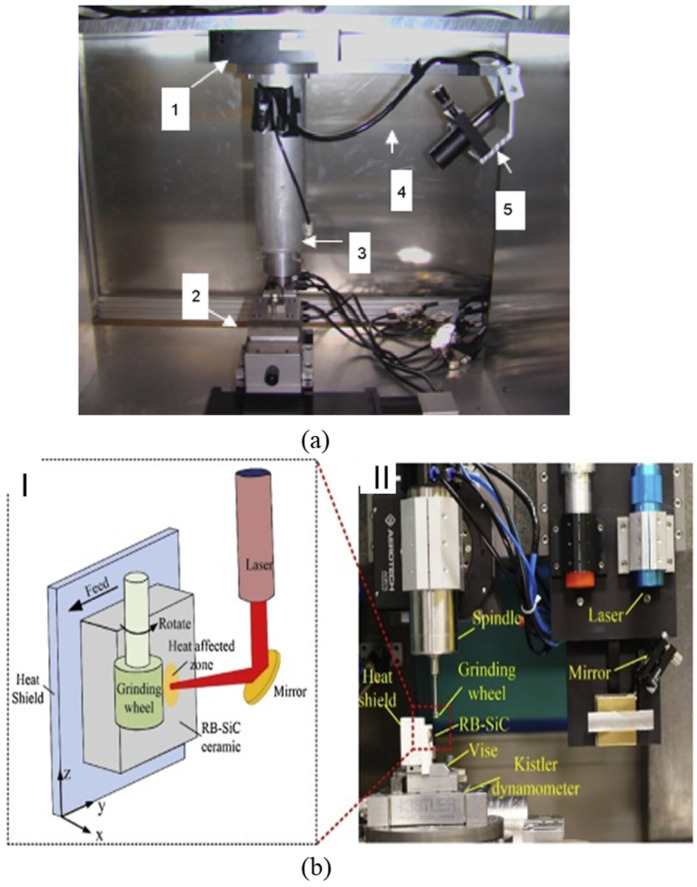
(**a**) Laser-assisted milling process experimental setup (1: rotary stage for orienting the laser, 2: stacked linear stages—X, Y and Z, 3: spindle assembly, 4: fibre optic cable, 5: collimator and micrometer assembly); adapted from [105]; (**b**) Laser-assisted microgrinding (I—schematic diagram; II—Experimental setup); adapted from [106].

For normal machining, LAM is carried out using a high-power laser which heats the BHM (workpiece) locally and selectively prior to the use of the traditional cutting tool to machine the material plastically [103,104]. The laser beams serve as a source of heat that changes the deformation mechanism from brittle to ductile without any phase change [104]. Laser-assisted machining processes that are employed to machine BHMs include laser-assisted turning (LAT), laser-assisted milling (LAM), laser-assisted grinding (LAG) at the microscale level, and single-point diamond turning at the nanoscale (Figure 11 and Figure 12).

Wang et al. [107] investigated the machining characteristics of SiCp/2024Al composite laser-assisted micro-machining (LAMM) to have an insight into chip formation, surface integrity, cutting force, and tool wear. This investigation reveals that the dominant component of cutting force was thrust force and this had a decrease of 27% when laser power increased from 0–31 W. The surface roughness was reported to have been influenced by the laser power and cutting depth, with the laser power contributing majorly. To achieve better surface quality, it is recommended that high laser power and small cutting depth should be employed. During LAMM of SiCp/2024Al, aluminium oxide nanoparticles were produced, and this was reported to have reduced the friction force between the cutting tool and the workpiece which resulted in reduced cutting tool wear. The conclusion on the tool wear by the authors was that the wear is more severe on the flank face than the rake face, and adhesion wear and abrasive wear play a significant role during LAMM. With an increase in laser power from 0–31 W, a 38% decrease in flank wear has been reported. Chang et al. [105] carried out laser-assisted grinding to investigate the effect of laser heating on the cutting force, grinding depth, and surface roughness when machining silicon nitride (Si_3_N_4_) and aluminium oxide (Al_2_O_3_). The investigation indicated that laser heating caused thermal expansion of these materials which led to deeper grooves, and a better surface roughness was obtained compared to the conventional grinding process and coolant-assisted grinding process. It is concluded that laser-assisted machining will be a useful machining technique to fabricate materials that are brittle like (Si_3_N_4_) and (Al_2_O_3_). Melkote et al. [106] investigated laser-assisted micromilling of hardened A2 tool steel and observed the following: there was less tool wear rate and consistency in surface roughness with lower value with laser heating than without it; throughout the whole cutting distance, the groove dimensional accuracy is superior. An investigation by Kannan et al. [108] shows that the surface temperature of alumina (Al_2_O_3_) during precision laser-assisted turning increases as the laser power increases but decreases with an increase in laser scanning speed. The conclusion is that with optimal laser parameters and machining parameters, there is a reduction in the cutting force, specific cutting energy, and tool wear compared to that without a laser. Joshi et al. [109] employed a finite element method to study the temperature distribution of LAM of the Ti-6Al-4V titanium alloy. They reported similar results as that of Kannan et al. [108] in that the workpiece’s surface temperature increases with laser power and decreases with an increase in spot radius and scanning speed.

However, LAM being a preheating process, excessive laser emission beams on the workpiece (material) surface may cause too much thermal stress and ablation. This may lead to crack generation on the surface and cause subsurface damage. Thus, an appropriate choice of laser parameters is essential to avoid surface damage to the workpiece because of excessive heat. It is reported that LAM is not suitable for ultraprecision manufacturing of optical moulds because the required surface roughness and shape accuracy is extreme, and the use of cutting fluid may be impossible. Thus, micro-laser-assisted machining (µ-LAM) is normally employed instead. It is established that micro-laser-assisted machining (µ-LAM) can apply cutting fluids simultaneously without interrupting the laser beam in addition to its thermal softening effect on the workpiece [63].

#### 4.5.2. Micro-Laser-Assisted Machining (µ-LAM) of Hard and Brittle Materials

**Figure 13:**
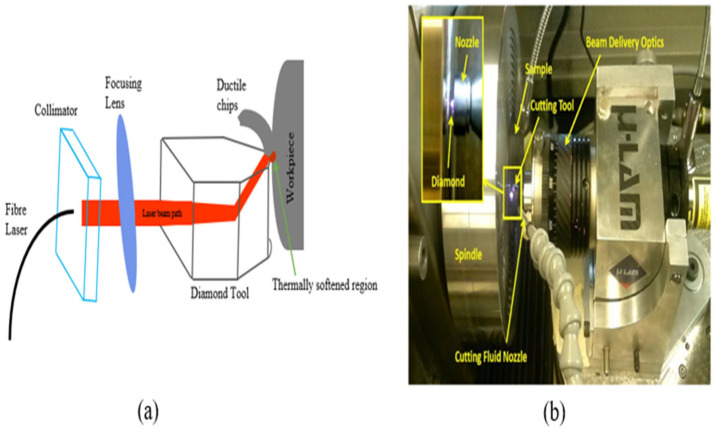
(**a**) Schematic overview of µ-LAM; (**b**) single-point diamond turning µ-LAM [63].

This is a hybrid machining technique that is different from the conventional LAM previously discussed in that it takes advantage of LAM by combining it with convectional ultraprecision machining. Micro-laser-assisted machining (µ-LAM) ensures that laser emission beams are focused on the tool-tip–workpiece cutting interface to enhance a more ductile cutting regime by preferentially heating and thermally softening the materials. The laser is positioned and adjusted to ensure that the laser beams pass through the diamond tool edge and that the laser absorption and the heating process are in the tool tip’s vicinity (Figure 13a). The hybrid SPDT and µ-LAM technique experimental setup is shown below (Figure 13b).

The research carried out by Patten et al. [110] reveals that the laser heating effect on nanomachining of silicon (Si) and silicon carbide (SiC) resulted in the enhancement of ductile response, greater cutting depths, a larger ductile–brittle transition, and smaller cutting forces. Results of numerical simulation of SiC after experimentation show that the material’s hardness decreases with an increase in temperature; and at elevated temperature, cutting forces, thrust forces, and pressures decreased. Ravindra and Patten [99] investigated micro-laser-assisted machining of three major polytypes of SiC, Si, and sapphire making use of single-point scratch tests and diamond turning and compared the results with machining without laser. The investigation makes known that laser-assisted cutting led to greater cutting depths, larger critical depth of cut, and lower cutting forces which in turn favours tool wear minimisation. This is so because the wear is a function of the resistance of the workpiece to cutting force; the lower the cutting force, the less is the possibility of the cutting tool becoming worn, and this is indicative of the good surface finish of the machined surface in term of surface roughness values.

**Figure 14:**
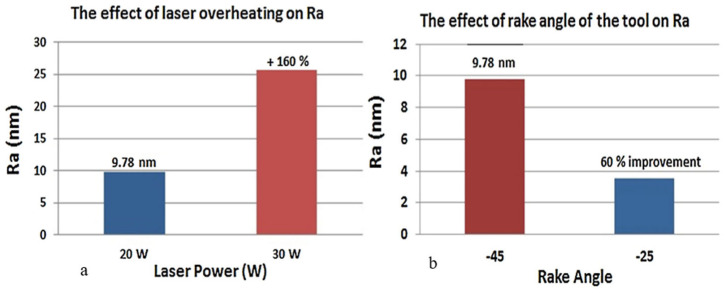
Effect of higher laser power on the machined surface finish (**a**) and (**b**) effect of highly negative rake angle on the machined surface finish [63].

Mohammadi et al. [63] investigated the effects of laser power, cross-feed rate, and tool rake angle on the surface finish of silicon using the hybrid method of SPDT and µ-LAM. The conclusion from this investigation is that it is possible to use the µ-LAM technique to obtain an optical-quality surface finish because the brittleness of the material decreases with laser heating and brittle fracture is avoided in the machining region. The effect of a highly negative rake angle on the machined surface was investigated by comparing the surface roughness of the rake angles of −45° and −25°. The silicon surface roughness after machining was reduced from 9.78–3.8 nm (60% improvement) with the tool using a rake angle of −25° and laser power of 20 W (Figure 14b). In their research, emphasis is placed on the laser power selection (optimal laser power of 20 W) because higher power could cause overheating, thermal cracks, and burning which would result in a rougher surface: 9.78 nm (20 W)–25.4 nm (30 W) surface roughness (Figure 14a). Recent research by Ke et al. [111] corroborates this statement. Ke et al. [111] observed that at a lower cutting speed of 500 mm/min, the resultant cutting force reverses by a sudden increase when the laser power exceeds 35.5 W as compared with the cutting speed of 1000 mm/min. The authors attributed this observation to the change in cutting mechanism from shearing to extrusion because of tool edge bluntness caused by material adhesion around the tool edge which, in turn, resulted in a high surface roughness value and led to an increase in the resultant cutting force. This material adhesion was claimed to be because of excessive high local temperature that caused overheating of the material workpiece. In comparison to conventional single-point diamond cutting of silicon with one coupled with µ-LAM, the critical depth of cut (DoC) increases by 364% [111]. It was concluded that the detriment to the cutting process is an excessively high laser power, and to obtain a large critical depth of cut, avoid overheating, a minimised resultant cutting force, and a small surface roughness, an optimised process parameter combination must be selected. Micro-laser-assisted machining (µ-LAM) has been used to fabricate or investigate the machinability of various materials with similar reports and observations stated herein [52,112,113].

### 4.6. Ultrasonic Vibration-Assisted Machining of Brittle and/or Hard Materials

An investigation of SiC internal grinding was carried out by comparing convectional internal grinding (CIG) and ultrasonic vibration-assisted internal grinding (UVAIG) [114]. The investigation reveals that the normal force and tangential force are reduced by 30.7% and 56.25% with UVAIG relative to CIG. After grinding, a decrease in surface roughness is observed: 76.5% (CIG) and 84.9% (UVAIG). Subsurface damages are reported to alleviate with a decrease of 17% of the fracture depth and reduced cracks through UVAIG. The material removal mechanism of polymer/carbon-fibre-reinforced composites (CFRP) was investigated by conventional scratching (CS) and ultrasonic vibration-assisted scratching (UVAS) through a rotary ultrasonic machining process suitable for hole-making and surface grinding of CFRP [115]. Comparing ductile-brittle transition (DBT), a longer ductile behaviour of 184 µm of DBT distance for UVAS while 54.32 µm of DBT distance was reported for CS at the same groove location. Scratching length beyond 54.32 µm led to fibre-matrix debonding, fibre breakage, and macro-cracks: an indicator of a brittle removal mode. For UVAS, fibre-matrix debonding and pull-out were reduced coupled with a large amount of material removal. However, a contrary report was provided by Wang et al. [116], who claimed there was a knowledge gap in the research on ultrasonic vibration-assisted machining (UVAM) of hard and brittle materials at the nanoscale. They investigated nanochannel machining on single-crystal silicon using a tip-based ultrasonic vibration-assisted scratching (UVAS) technique to gain an insight into its material removal mechanism and subsurface damage while undergoing ductile machining. The investigation was carried out experimentally and with a simulation-based method using molecular dynamics by comparing static scratching (SS) and UVAS. It was concluded that nanoscale UVAM induced deeper subsurface damage compared with UVAM at the micro-scale and convectional static machining. Therefore, it is suggested that the choice of UVAM may not be okay to suppress subsurface damage of hard and brittle materials (such as silicon) particularly when the depth of machining approaches the nanoscale.

### 4.7. Sustainability in Precision and Ultraprecision Manufacturing

**Figure 15:**
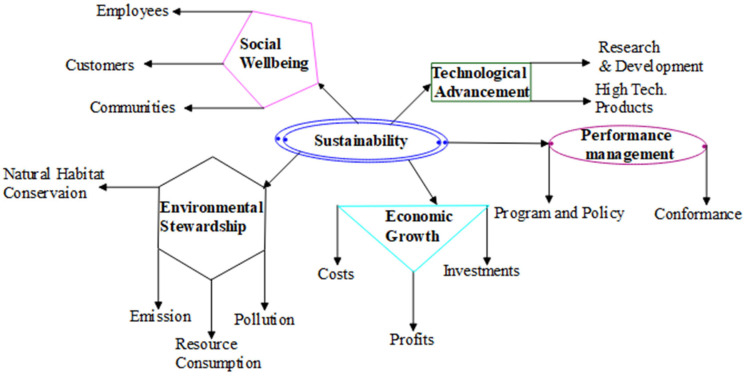
Crucial factors for consideration in sustainable manufacturing [118].

It is a fact that ultraprecision or precision manufacturing processes are the main technologies that are at the forefront of ensuring meeting up with rising demand in micro parts and components. It is therefore necessary to ensure their sustainability. Conservation of the environment is a major call by everyone across the world. Manufacturing industry activities directly or indirectly contribute to environmental degradation either pollution by air, land, or sea. More than three decades ago, the whole world was clamouring for zero emissions, green manufacturing, circular economy, or environmental sustainability; machinists or machining researchers are no exception to this view. Sustainability in manufacturing has been categorised majorly into three dimensions, environment, economic, and social, which are referred to as a triple bottom line that emphasises the importance of an organisation to prepare for three different bottom lines, the three Ps: profit, people, and planet [117]. However, sustainability is categorised into five dimensions with the inclusion of technological advancement and performance management in the abovementioned three dimensions (Figure 15) [118]. These five dimensions are good indicators that are crucial factors for sustainable ultraprecision/precision machining.

#### 4.7.1. Sustainable Techniques in Mechanical Machining

**Figure 16:**
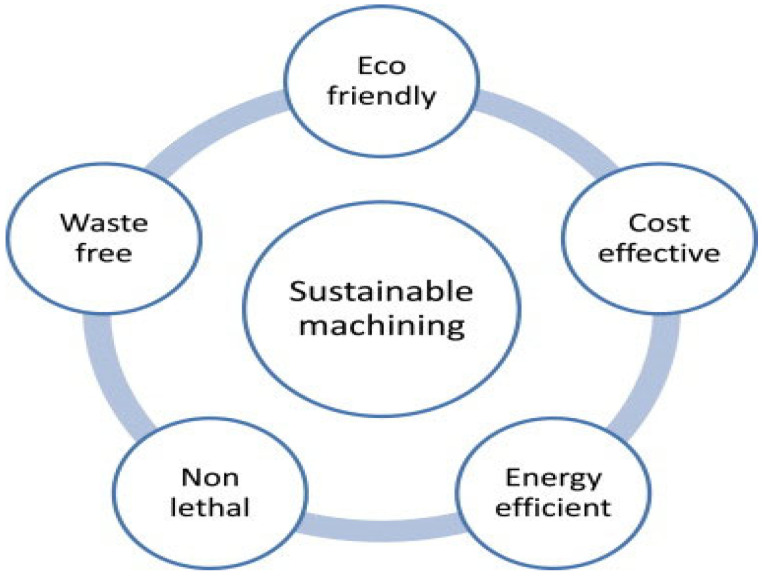
Characteristics of sustainable machining [124].

**Figure 17:**
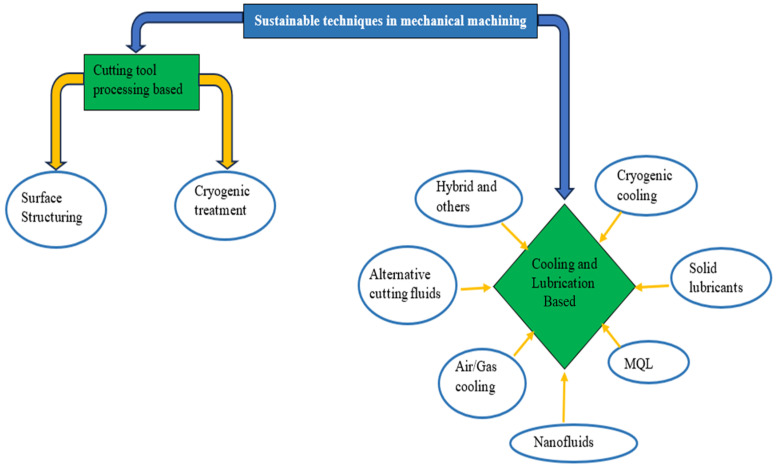
Sustainable manufacturing techniques for cleaner production: modified from [124].

The ultraprecision or precision mechanical machining processes discussed previously as a manufacturing process have contributed to sustainable manufacturing by ensuring minimal waste generation. Sustainable machining has been viewed to be aiming at eco-friendliness, cost-effectiveness, energy efficiency, illness-free operation, and being waste-free (Figure 16). Ultraprecision or precision mechanical machining of BHMs requires cutting fluid to ensure prolonged cutting tool life (less tool wear), improved surface finish, reduced cutting temperature, reduced production costs, and more. These fluids perform these major functions in machining: lubrication, cooling, chip removal, and corrosion protection [119,120]. However, cutting fluid has its disadvantages: negative impacts on the environment and human health, and high cost of storing and disposal [117]. Answering the call for sustainability in machining processes, the researchers in this discipline have performed a series of investigations that include dry cutting, reduction in power consumption, alternative cooling/lubrication techniques to conventional cooling/lubrication techniques (mist cooling, flood cooling, and high-pressure cooling), hybrid cooling/lubrication techniques, replacement of mineral oils/petroleum-based oils with nontoxic and biodegradable oils (vegetable oils), and others (Figure 17) [121,122,123,124]. Each of these sustainable machining techniques (Figure 17) has its pros and cons, and these are stated by Chetan et al. [124].

#### 4.7.2. Minimum Quantity Lubrication (MQL)

**Figure 18:**
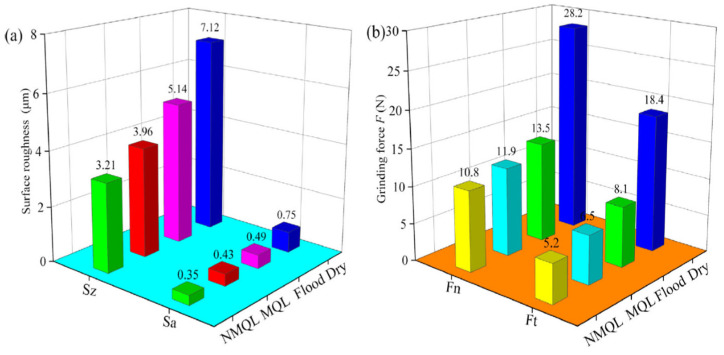
Influence of lubrication conditions on (**a**) the surface roughness and (**b**) the grinding force of C_f_/SiC at C = 5 g/L, P = 7 bar, Q = 80 mL/h, L = 60 mm [126].

**Figure 19:**
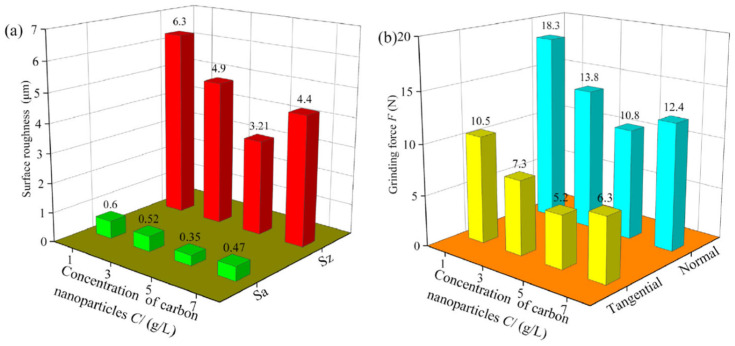
Influence of concentration of carbon nanoparticles C on (**a**) the surface roughness and (**b**) grinding force of C_f_/SiC composites at P = 7 bar, Q = 80 mL/h, L = 60 mm [126].

**Figure 20:**
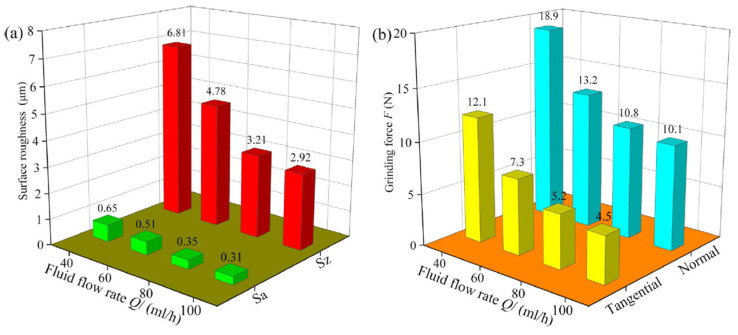
Influence of the fluid flow rate Q on (**a**) the surface roughness and (**b**) grinding force of C_f_/SiC composites at C = 5 g/L, P = 7 bar, Q = 80 mL/h, L = 60 mm [126].

Minimum quantity lubrication (MQL) has been reported to be an effective technique for cutting performance enhancement in precision mechanical and ultraprecision mechanical machining [125,126]. The low cooling effect has been identified as the main drawback of the MQL technique, so it is not fit for cutting BHMs with high strength and hardness. To address this drawback, recent researchers employ nanofluids (suspended nanoparticles like diamond, CuO, graphene, and others to the base fluids) while applying the MQL technique to improve the cutting performance and productivity [126,127,128,129]. An investigation of the effects of dry, flood, MQL, and carbon nanofluid MQL (NMQL) conditions on grinding performance of unidirectional carbon-fibre-reinforced composites of an SiC matrix reveals the capability of carbon NMQL conditions to provide a significantly improved surface quality, minor surface damage, and reduced grinding forces compared to dry, flood, and MQL conditions, and safety of the environment and workers are ensured by using less grinding fluid which in turn helps in ecological environment protection (Figure 18) [126]. The effects of the parameters of carbon NMQL (particle concentration, flow rate, air pressure, nozzle position, and nozzle distance) were also investigated. The effects of both carbon concentration (C) and fluid flow rate (Q) on surface roughness and grinding forces are illustrated in Figure 19 and Figure 20, respectively.

The optimum carbon concentration and fluid flow rate are found to be 5 g/L and 80 mL/h, respectively, and as the fluid flow rate increases, both surface roughness and grinding forces decrease, which implies improved surface quality and improved lubrication performance, respectively.

**Figure 21:**
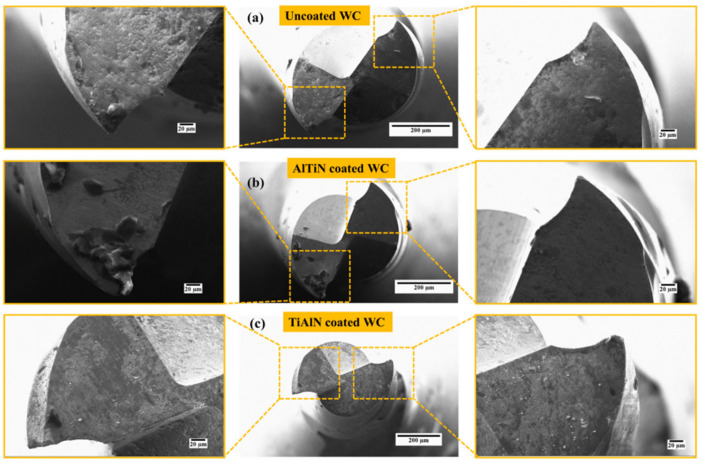
SEM images of (**a**) uncoated, (**b**) AlTiN-coated, and (**c**) TiAlN-coated WC micro end-mills in nano-MQL conditions with 1 vol% CuO after 450 mm cutting length [127].

**Figure 22:**
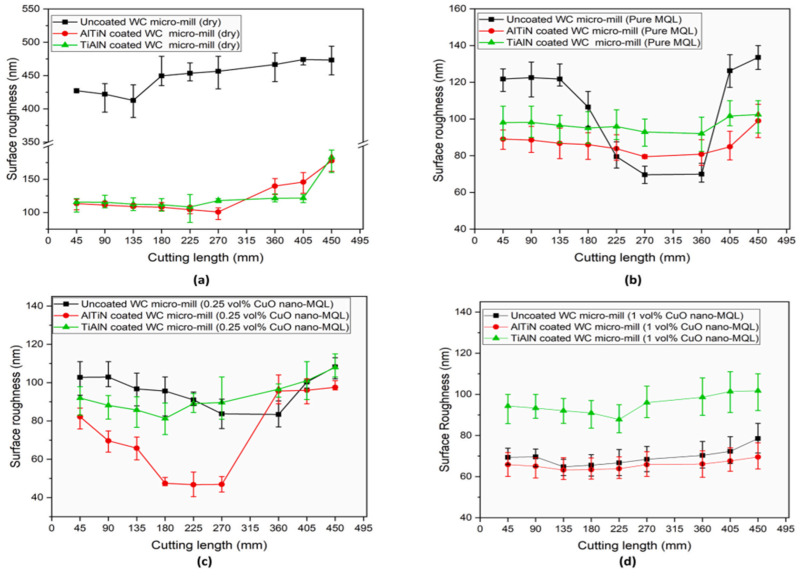
The variation of average surface roughness with machining length by uncoated, AlTiN-coated, and TiAlN-coated WC micro end-mill in (**a**) dry, (**b**) pure MQL, (**c**) 0.25 vol% CuO nanofluid MQL, and (**d**) 1 vol% CuO nanofluid MQL conditions [127].

An investigation to know how tool life longevity and surface quality can be improved was carried out by Roushan et al. [127] via a hybrid MQL technique using deionised water and CuO nanofluid with PVD-coated (AlTiN/TiAlN) and uncoated WC micro end-mills in micromilling of Ti-6Al-4V alloy under various environmental conditions. Under these conditions, tool wear in the form of abrasion, adhesion, coating delamination, grain pull-out, edge chipping, and built-up edge was observed. The cutting environment that provided no indication of any form of tool wear is one with an increased nanoparticle concentration (1 vol% of CuO nanofluid MQL conditions) and TiAlN-coated WC (Figure 21c). Also, under these different cutting environmental conditions and with cutting length variation, the surface roughness with the lesser value and less significant variations in nearly all conditions is that of 1 vol% CuO nanofluid MQL (Figure 22d). Also, 0.25 v% CuO nanofluid MQL with AlTiN coating displays an excellent surface quality with some surface roughness of less than 50 nm (Figure 21c). These observations are attributed to the adhesion absence and cutting tool edges, which remained unworn, and improved wettability as well as maintaining cutting edge sharpness because of the higher concentration of nanoparticles. The overall conclusion from this investigation is that significant tool life and surface quality improvements are achievable by employing a hybrid MQL technique and coated tools compared to a singular application of coated tools or MQL techniques during micro-milling of BHMs such as Ti-6Al-4V alloy.

Similar observations were previously reported by Sinha et al. [128]. Their investigation reveals that under different environmental cutting conditions of dry, wet, small quantity lubrication (SQL) with soluble oil, and SQL with nanofluids of silver and zinc oxide nanoparticles (believed to be environmentally sustainable) in deionised water, application of nanofluids with the SQL technique while grinding Inconel 718 produced minimal grinding forces and coefficient of friction with a better ground surface integrity. It was concluded that zinc-oxide-based nanofluids produced better grinding responses due to the induction of stable lubricious film at the contacting surfaces more exactly at a higher temperature because of better spreadability [128].

An investigation performed by Huang et al. [130] to gain insight into the cutting performance of titanium (Ti-6Al-4V) by carrying out micro-scale diamond turning at different cooling conditions of oil-based MQL, cryogenic gas (CG), and hybrid MQL and CG revealed that, apart from low lubricity of hybrid CG and MQL due to low temperature that resulted in noticeable tool wear and material adhesion (less pronounced in MQL cooling), hybrid CG and MQL is capable of achieving improved surface roughness, minimal cutting fluid consumption, and lesser environmentally and occupationally hazardous effects. This implies that the CG and MQL cooling condition has the highest cooling efficiency. The low lubricity effect of CG and MQL has given rise to recommendations for future studies. Ibrahim et al. [122] investigated the effectiveness of palm-oil-based nanofluids employing graphene nanoplatelets (GNPs) of various concentrations (0.1–0.4 wt%) using the MQL lubrication technique to carry out cutting and tribological and microstructural tests on titanium (Ti-6Al-4V) alloys. Acculube LB2000 MQL cutting fluid was used as a reference oil and a ZrO2 ball was employed as the grinding tool. The observations that emerged from this investigation include the following: the nanofluid with 0.1 wt% of GNPs lessen the specific grinding energy by 91.78% compared to dry cutting; in comparison with Acculube LB2000, nanofluid with 0.1 wt% GNPs provided a reduction of about 80.25% of specific cutting energy (SCE) due to extreme reduction in the friction coefficient; overall, the palm-oil-based nanofluids/MQL when compared with dry, flood, and LB200/MQL modes produced a relatively improved surface quality of the ground surfaces, and it is eco-friendly. An investigation to enhance sustainability and improve the performance of conventional grinding (CG) was conducted by Singh and Sharma [131] by utilising the dual advantages of ultrasonic-assisted grinding (AUG) and ultrasonically atomised cutting fluid (UaF) (ionic-liquid-based rice bran oil). After a comparison of output parameters like cutting force and surface integrity for different grinding conditions of CG, AUG, UaFCG, and UaFAUG, the product sustainability index (PSI) was evaluated after a life cycle analysis (LCA) of the grinding processes was conducted by the same authors. The subcomponents (indicators) and cluster of the PSI study (Table 2) revealed that the UaFAUG technique is 46.06% more sustainable than the CG process of Nimonic 80 A, which in turn is an indication of sustainable grinding processes. It was reported that the UaFAUG method produced a noticeable reduction in normal force, tangential cutting force, coefficient of friction, and surface roughness by 66.22%, 52.66%, 30.42%, and 46.48%, respectively, as compared to CG [131].

### 4.8. Ultraprecision Mechanical Machining: Molecular Dynamics Simulation

The role of modelling and simulation in manufacturing processes cannot be ruled out in the sustainable ultraprecision mechanical machining of BHMs and the production of components for MEMS devices because of the exorbitant cost of the experimental setup, environmental sustainability, and ecosystem management. As a result, some investigations involving molecular dynamics modelling and simulation of some precision mechanical machining processes of some BHMs are reviewed.

**Figure 23:**
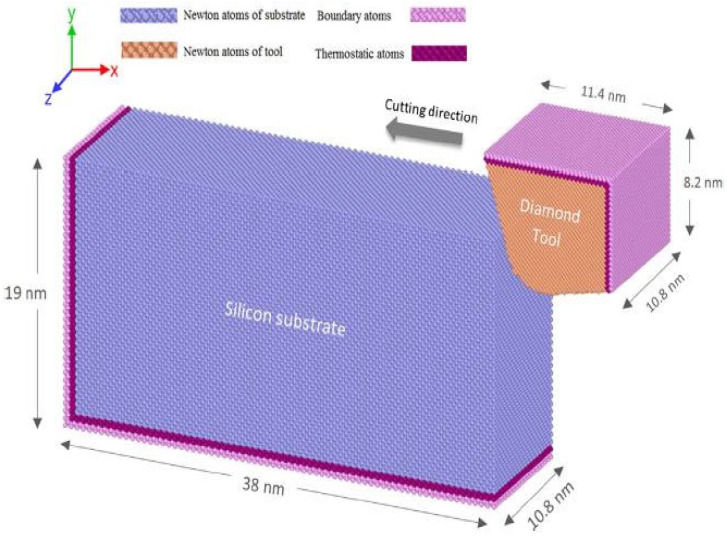
Nanometric cutting: MD simulation model, adapted from [136].

Molecular dynamics is commonly employed as a simulation tool for precision or ultraprecision machining. Very little research is carried out using the finite element method (FEM) [132]. The reason is that finite element analysis is not good enough to observe the complex phenomena displayed when ultraprecision machining is being carried out [133]. The schematic molecular dynamics (MD) simulation model of a nanometric cutting is illustrated below (Figure 23). Shimada et al. [134] used single-crystal silicon Si and LiNbO_3_ to perform micromachining (turning, grinding, and indentation) experiments and MD micro-indentation and cutting simulations to see how applicable the hypothesis of brittle–ductile transition (BDT) phenomena is. They concluded that brittle materials can be machined in ductile mode down to nanometric or near-nanometric levels, regardless of how brittle they are. Goel et al. [135] carried out single-point diamond turning via MD simulation to investigate the diamond tool wear mechanism of single-crystal silicon. It was reported that ductile machining of silicon occurs at the cutting zone because of the formation of the metallic phase of silicon (Si-II) brought about by high-pressure phase transformation (HPPT). The authors claimed that the formation of silicon carbide (tribochemistry) and the sp^3^-sp^2^ disorder of diamond are the main wear mechanisms of diamond cutting tools during SPDT of silicon.

Wang et al. [137] employed MD simulation to understand the effect of crystal anisotropy on the nanometric cutting of single-crystal silicon. The investigation classified the chips into amorphous chips whose formation mechanism was attributed to the presence of high-pressure phase transformation and amorphous crystallite chips whose formation mechanism was due to the combined effect of high-pressure phase transformation and cleavage. In comparison with other directions on the same crystal plane, the (100)[1–10], (110)[1–10], and (111)[1–10] directions are said to be more fit for ductile cutting of silicon. Cai et al. [138] used the MD simulation method to examine the mechanics behind monocrystalline silicon wafer nanometric cutting in the ductile regime. These two criteria, it is said, must be met for the transformation from brittle to ductile machining to take place:

(a) Undeformed chip thickness must be less than the tool edge radius.
(b) The radius of the tool cutting edge should be so small that this can be maintained.

Xiao et al. [139] carried out an MD simulation of brittle-ductile cutting mode transition by taking silicon carbide as a case study and focusing on the undeformed chip thickness. There was a transition from ductile-mode cutting to a mixed mode of ductile chip formation and brittle fracture, and brittle-mode cutting as undeformed chip thickness increased as revealed by the MD results. It was also reported that an increase in the undeformed chip thickness led to an increase in the tensile stress around the cutting zone until the critical undeformed chip thickness was exceeded, which eventually led to brittle fracture [139]. The MD results indicated that crack formation location and direction of propagation vary with undeformed chip thickness.

MD simulation has been used to investigate laser-assisted machining of some BHMs such as silicon. MD simulation is employed to investigate the effects of laser parameters such as laser spot radius, laser speed, and laser pulse intensity on the mechanism of material removal and subsurface damage (SSD) of silicon (monocrystalline) during high-speed laser-assisted grinding of the silicon [140]. Reduced friction coefficient, improved material removal rate, and grinding depth increase were reported as the laser pulse intensity increased. However, a smaller laser pulse intensity was recommended to avoid thermal deformation of the workpiece. Grinding forces decrease and grinding depth reduces as the laser moving speed increases and the laser spot radius increases, respectively [139]. Reduction in the grinding depth for a larger laser spot radius was attributed to a decrease in the energy per unit area in the laser irradiation zone on the workpiece surface. Also, to ensure an improved material removal rate, an optimal laser spot radius (3 nm for the case study) should be chosen [139]. An investigation using molecular dynamics simulations of traditional grinding and laser-assisted grinding of gallium nitride (GaN) by Li et al. [141] concluded an excessive laser power density resulted in excessive damage to the abrasive rake face and this led to a reduction in the machined surface finish quality. Thus, an appropriate laser power density below 1.5 × 10^9^ W/cm^2^ is recommended for an effective reduction in wheel wear and an increase in grinding efficiency when grinding single-crystal GaN.

## 5. Challenges/Limitations

In process optimisation, the effort is to achieve the required precision by optimising machining parameters. Balancing machining parameters while minimising waste and energy use is challenging. Ultraprecision machining of composites is even more complicated than other materials because of the presence of reinforcement, and major interaction at the interface of the tool and workpiece becomes more challenging. Achieving consistency in the machining result of composites is challenging because of their anisotropic properties wherein reinforced fibre orientations play major roles. The minimum quantity lubrication (MQL) method coupled with nanofluids has been reported as suitable for sustainable ultraprecision machining of BHMs. The stability of the nanoparticles in the base fluid is difficult to maintain, as they tend to settle and agglomerate over time. This behaviour does lead to non-uniform distribution and reduced performance. Determining the optimal concentration of nanoparticles in the base fluid is another challenge. The major challenges with the use of coolant and lubricant in ultraprecision machining of BHMs are health and safety and environmental impacts. Sustainability in manufacturing is a good initiative; however, balancing sustainability with cost-effectiveness becomes an issue for hugely easy adoption in the industry.

## 6. Outlook and Perspective of Mechanical Ultraprecision Machining Research

The following recommendations for future research in mechanical ultraprecision machining are suggested:

i. Implementation of in-process monitoring techniques and control, like acoustic emission and vibration sensing, that could ensure real-time detection and prevention of machining-induced damage is highly needed.
ii. Intensify research efforts on hybrid machining processes such as laser-assisted machining; ultrasonic vibration-assisted machining; and high-pressure coolant-assisted machining. Ultraprecision machining could be integrated with additive manufacturing methods to create hybrid components with engineered properties and functionalities.
iii. Research efforts should be made to determine the optimisation of nanofluid performance and develop advanced formulations, and the best principles of their use in ultraprecision machining of brittle and hard materials are established.
iv. Implementation of MQL techniques and investigation of the environmental impact and life cycle assessment.
v. Research and development efforts should be geared toward potential health and safety associated with the use of nanofluids in machining processes.
vi. Investigating the incorporation of sustainability metrics into MD simulations to predict the environmental impacts of various machining processes could improve sustainable manufacturing.
vii. Sensors that can monitor energy consumption during various machining techniques are recommended. If highly sensitive sensors are available, energy consumption can be reduced, and this enhances the sustainable machining of BHMs.

## 7. Conclusions

Based on the review and analysis of mechanical ultraprecision machining conditions and some major influencing parameters, the following conclusions can be drawn:

The technologies of ultraprecision machining have improved, and this ensures that surface integrity and a surface finish of high quality are achievable. However, there is a need to address some challenges like tool wear, how exact surface integrity can be achieved, applying appropriate ductile–brittle transition mechanisms, optimisation of machining parameters and control processes, and so on. In addition to the high cost of equipment set-up for ultraprecision machining techniques, these challenges are contributing factors that prevent the mass production of components. To address this issue, collective collaborative research among mechanical and materials engineers, physicists, materials scientists, nanotechnologists, and so on is necessary.

In addition, composites are now attracting the attention of machining researchers because of their wide applications, especially in areas where desired and suitable properties need to be engineered. Putting more research efforts into addressing the challenges confronting ultraprecision machining of composite materials by taking advantage of the latest advancements in ultraprecision machining will ensure the production of high-quality and precise components. It is also apparent that sustainable manufacturing is now new terminology before and since the call for a zero-emission campaign. Dry or near-dry machining is currently a focus of machinists or machining researchers, and MQL with nanofluids is viewed as a promising principle that improves cutting performance and reduces environmental impact. Hybrid techniques by combining MQL with other methods such as cryogenic cooling have shown promise in improving surface quality and reducing tool wear. Molecular dynamics modelling and simulation have been employed to address some of the challenges associated with the ultraprecision machining of BHMs. The development of numerical models for ultraprecision machining has advanced predictive capabilities and this has brought about a reduction in relying on costly experimental trials. However, the possibility of incorporating sustainability metrics into MD simulations to predict the environmental impacts of various machining processes stated herein could help in developing more eco-friendly machining processes.

Research on the development of novel lubrication and environmentally friendly cooling methods would advance the field of ultraprecision machining and tribology. Research on sustainable ultraprecision machining would contribute to reducing the ecological footprint of precision manufacturing processes. The development of less hazardous cutting fluids and techniques would address occupational health concerns, and the investigation of bio-based and ionic liquids as cutting fluids would promote the use of renewable resources.

## Figures and Tables

**Table 1:** Fabrication techniques of diffractive micro/nanostructures on IR materials.

Fabrication Technique Types	Fabrication Techniques	Limitations
Mechanical fabrication	Ultra-precision diamond cutting; diamond milling, fast tool servo (FTS), slow tool servo (STS), and vibration-assisted cutting	The height variation of the generated hybrid IR surfaces that is achievable is limited due to the adoption of very small feed rates and depths of cuts (FTS/STS). There is a restriction to the highest attainable periodicity of the secondary diffractive micro/nanostructures (FTS/STS).
	Ultrasonic vibration-assisted diamond cutting	Only suitable for planar surfaces. Difficult to generate hybrid structures with a freeform primary surface.
Non-mechanical fabrication	Electron beam lithography, laser ablation, focused ion beam	Restriction to planar substrates. They cannot generate concave/convex surfaces with a shorter radius of curvature.
	Etching (laser-assisted and chemical etching)	Facility requirements are expensive. Difficulty of generating complex secondary structures.
	Femtosecond laser polymerisation	Restriction to low-efficiency and photocurable polymer materials.
Hybrid (Mechanical and non-mechanical)	Ultra-precision machining and picosecond laser ablation	Introduction of unnecessary machining errors. Efficiency is relatively low, and it is relatively costly.

**Table 2:** Subcomponents and clusters of PSI study (adapted from [131]).

Subcomponents	Clusters
Economy	Material cost Cutting fluid cost Electricity cost
Environment	Global warming Ozone depletion Fine particulate Matter formation Water consumption Acidification, eutrophication, and ecotoxicity Carcinogenic and noncarcinogenic Resource scarcity
Societal	Employee safety Human health
